# How Many Dystonias? Clinical Evidence

**DOI:** 10.3389/fneur.2017.00018

**Published:** 2017-02-03

**Authors:** Alberto Albanese

**Affiliations:** ^1^Department of Neurology, Humanitas Research Hospital, Milan, Italy; ^2^Department of Neurology, Università Cattolica del Sacro Cuore, Milan, Italy

**Keywords:** dystonia, movement disorders, history, definition and concepts, phenomenology

## Abstract

Literary reports on dystonia date back to post-Medieval times. Medical reports are instead more recent. We review here the early descriptions and the historical establishment of a consensus on the clinical phenomenology and the diagnostic features of dystonia syndromes. Lumping and splitting exercises have characterized this area of knowledge, and it remains largely unclear how many dystonia types we are to count. This review describes the history leading to recognize that focal dystonia syndromes are a coherent clinical set encompassing cranial dystonia (including blepharospasm), oromandibular dystonia, spasmodic torticollis, truncal dystonia, writer’s cramp, and other occupational dystonias. Papers describing features of dystonia and diagnostic criteria are critically analyzed and put into historical perspective. Issues and inconsistencies in this lumping effort are discussed, and the currently unmet needs are critically reviewed.

## Literary Descriptions of Dystonia

Dystonia has been defined by Denny-Brown as “the most striking and grotesque of all neurological disorders” (1). Therefore, it is not surprising that artists have reported the feature of dystonia before doctors were able to categorize its striking phenomenology. Cervical dystonia, the most prevalent dystonia type, has been the object of some famous literary portrayals. The first artistic description dates back to 1315, when Dante Alighieri reported having seen fortune tellers and diviners punished in the Inferno (Circle eight, *Bolgia* four), by the divine law or retaliation for having looked too far forward, with their head twisted backwards (2):

And when I looked down from their faces, I saw

that each of them was hideously distorted

between the top of the chest and the line of jaw;

for the face was reversed on the neck, and they came on

backwards, starting backwards at their loins,

for to look before them was forbidden. Someone

sometime, in the grip of palsy may have been

distorted so, but never to my knowledge;

Rabelais (circa in 1532) introduced the French neologism torticollis (*torty colly* in the original old French). Into a similar infernal atmosphere, he described the healing of Epistemon, “who had his head cut off, was finely healed by Panurge, and brought news from the devils, and from the damned people in hell” … “Thus as they went seeking after him, they found him stark dead, with his head between his arms all bloody” … “Panurge took the head and held it warm foregainst his codpiece, that the wind might not enter into it. Eusthenes and Carpalin carried the body to the place where they had banqueted, not out of any hope that ever he would recover, but that Pantagruel might see it. … Then cleansed he his neck very well with pure white wine, and, after that, took his head, and into it synapised some powder of diamerdis, which he always carried about him in one of his bags. Afterwards he anointed it with I know not what ointment, and set it on very just, vein against vein, sinew against sinew, and spondyle against spondyle, that he might not be torticollis (for such people he mortally hated). This done, he gave it round about some fifteen or sixteen stitches with a needle that it might not fall off again; then, on all sides and everywhere, he put a little ointment on it, which he called resuscitative.”

Other artists have described focal dystonias, particularly cervical dystonia, but not as much the generalized cases, that may have appeared too severe to become a narrative subject.

## Early Medical Descriptions

It is of interest to follow the historical order by which different dystonia types were recognized and described. It is also remarkable to note how some fundamental clinical questions have remained actual, and for the most unanswered, until now.

### Cervical Dystonia

Cervical dystonia has attracted early medical interest. Tulpius (3) gave one of the first descriptions of torticollis; he considered a contraction of the scalene muscles as the most common cause. A first classification was attempted by Heister (4), who distinguished “caput obstipum” from “collum obstipum,” a phenomenological distinction that has been recently proposed anew (5). A prominent role of the sternocleidomastoid muscle was later widely recognized and surgical sections of its tendons started being performed by orthopedic surgeons. A non-surgical approach, based on head repositioning under anesthesia followed by head bandage, later gained diffusion in France and abroad as an alternative to surgical ablations (6). This approach attracted a wide medical audience interested in the management of “muscular torticollis” (then distinguished from torticollis caused by scars or bone anomalies; Table 1). The monumental medical encyclopedia edited by Dr. Fabre reported: “It appears today that the majority of neck muscles may become the starting point of a permanent retraction and may also contribute to some type of head deviations” (7). The complex phenomenology of torticollis was also recognized: “the sternocleidomastoid is not the only muscle that may be involved; the majority of other cervical muscles may be involved, so to produce the attitude of torticollis either by their specific action or by a combined influence” (8). Torticollis became then a matter for neurologists. Pitres recognized that there was no pathognomonic sign to distinguish spasms of hysteric origin from the non-hysteric ones (9). The non-hysterical nature of torticollis was reinforced by the observation that patients with torticollis also had “functional spasms” in other body regions (10).

**Table 1:** **Classification of cervical dystonia by the end of nineteenth century (11)**.

Etiology	Cicatricial torticollis Muscular torticollis Articular (or osseous) torticollis	Retraction of skin and subcutaneous tissue following burn, abscess, phlegmon, etc. Muscular abnormality caused by contraction, retraction (often congenital), paralysis, and spasm (intermittent or spasmodic) Abnormality of joints or cervical vertebrae

Onset	Congenital Acquired

Phenomenology	Anterior Posterior Lateral

*Cicatricial torticollis was considered very common, as it was often observed in soldiers returning from battlefields*.

### Generalized Dystonia

Generalized dystonia was listed for the first time by Gowers under the name of “tetanoid chorea,” one of the several choreatic disorders encompassing also senile chorea, maniacal chorea, functional chorea, and Sydenham’s chorea (12). He had probably also observed idiopathic generalized cases, but later defended that “tetanoid chorea” was a feature of Wilson’s disease (13).

Undoubtedly, 1911 is the founding year of generalized dystonia as an independent nosological entity. Hermann Oppenheim, probably the most famous German neurologist of the time, published the description of *dystonia musculorm deformans* (altered muscle tone causing deformities), a condition he observed in four unrelated Jewish children who came to Berlin to be seen by him (14). He also added a second Latin descriptor *dysbasia lordotica progressiva* (progressive gait difficulty with lordosis), as he noted the occurrence of pronounced and progressive lordosis in his patients. A similar phenomenology had been previously observed in a Jewish kindred by Theodor Ziehen, professor of psychiatry in Berlin, who asked his resident Markus Walter Schwalbe to write a dissertation on this peculiar phenomenology. Ziehen presented his observations in Berlin in December 1910 and published the family in 1911 as well (15). Finally, Edward Flatau and Wladyslaw Sterling described the same condition observed in two Polish Jews under the name of “progressive torsion spasm” in 1911 as well (16).

Unquestionably, Oppenheim’s publication is the most prominent of the three. Patient 1 described by Flatau and Sterling was also seen by Oppenheim himself, and Flatau and Sterling repeatedly mention Oppenheim’s seminal publication. Both Oppenheim and Flatau and Sterling underline the organic nature of the disease and reject Schwalbe–Ziehen’s label of hysterical disorder. Their reasoning is however different. Oppenheim considered the phenomenology of Schwalbe–Ziehen’s cases different from that of *dystonia musculorum deformans* he was describing. He reported: “I find profound differences between these observations and those of my own, also with respect to the account of this affliction given by Ziehen himself. Hence, Schwalbe describes choreiform and tic-like movements.” Flatau and Sterling, instead, used clinical arguments to reject the hysterical nature of the phenomenology they had observed. They reported: “The first boy we first saw in 1909 offered us great diagnostic difficulties. Some colleagues, to whom we presented the case in the hospital, thought of hysteria. We have, however, denied this diagnosis in a more precise analysis, and considered the case as an unknown form of spasm. The longer we have observed the patient, the deeper was the conviction that we were not dealing with any functional disease, but with a disease of its own” … “The clinical picture was so characteristic that when one of us saw the second case in his office, he immediately thought of his similarity with the first.”

The clinical descriptions in Flatau–Sterling publication are particularly interesting, probably because they have appeared later than Oppenheim’s publication. Oppenheim defended that: “the clonic jerks do indeed belong to the clinical picture,” “the hypotonia is a major element of the symptomatology,” and “the tonic cramps are very predominantly connected with the function of standing and walking.” Hence, he proposed to name the disease “dystonia” (i.e., abnormal muscle tone) or “dysbasia” (i.e., abnormal base while standing or walking). Flatau and Sterling reasoning led them to dispute this terminology. They stated: “With the name suggested by Oppenheim (*Dysbasia lordotica progressiva* and *Dystonia musculorum deformans*), we cannot be satisfied for the reason that in some, such as our two patients, the disease is as strong in the upper as in the lower extremities, and dysbasia is not the principal symptom. We have also shown that there is no hypotonia in our patients, and we also believe that the word ‘deformans’ contains something stable, which is not true in the case of the essentially mobile spasm.”

Numerous reports followed. In a review of all published cases, Mendel summarized the significant clinical data and introduced the expression “torsion dystonia” to indicate a specific nosologic entity distinguished from other types of involuntary movements, such as hysteria, double athetosis, chorea, and myoclonus (17). During the following decades, different types of hyperkinetic movements (including dystonia) were reported in patients with encephalitis lethargica, Wilson’s disease, or cerebral palsy (then called double athethosis). The issue whether dystonia was a disease entity or instead a syndrome of basal ganglia dysfunction with different possible causes arose. Dystonia became the topic of some dedicated symposia where similarities between focal and generalized dystonia types were mentioned. At the 95th Annual Meeting of the British Medical Association held in Edinburg in 1927, a session was devoted to the “existing confusion on involuntary movements.” Guillain there defended the view that torticollis was not a psychogenic condition (18). In 1929, a session of the Tenth French Congress of Neurology was devoted to “torsion spasms,” with lively discussions about their psychiatric vs. organic origin (19). This issue was gradually settled by the observation that torticollis and torsion spasms (initially defined as pseudo-parkinsonism) could be secondary to encephalitis lethargica (20). In 1940, the Association for Research in Nervous and Mental Diseases extensively discussed the phenomenology of dystonia, its similarity with athetosis, the related EMG reading, and the underlying pathology (21). According to Herz (21), the term *dystonia musculorum deformans* should be confined to the idiopathic form. He gave the following criteria for the clinical diagnosis of idiopathic dystonia: (a) selective systemic symptoms in the form of dystonic movements and postures; (b) gradual development, without recognizable etiological factors at the onset. He also distinguished early forms occurring shortly after birth, from the juvenile form with the onset between 5 and 15, and the late form after 15 years of age.

### Other Focal Dystonias

Notwithstanding these scholarly observations, torticollis and generalized dystonia were still considered two separate and distinct conditions. Craft neuroses (also called occupational spasms or trade palsies) were also considered a distinct group of “functional disorders which are characterized by a difficulty in performing specific coordinated movements of certain occupations” (22). The best known craft neuroses were writer’s cramp and telegraphists’ cramp.

Writer’s cramp was another medical condition known since 1700 from the work of Bernardino Ramazzini, the father of occupational medicine (23), who stated: “The diseases of persons incident to this work arise from three causes; firstly, constant sitting, secondly the perpetual motion of the hand in the same manner, and thirdly the attention and application of the mind. Constant writing considerably fatigues the hand and whole arm on account of the almost continual and almost tense tension of the muscles and tendons.” In the mid-nineteenth century, Duchenne (24) and Gowers (25) wrote extensively about writer’s cramp. Solly provided an early surgeon’s view and considered writer’s cramp a spinal cord disorder (26). The largest early series was published by Poore (27), whose classification was based on tenderness and measures of faradic response. A comprehensive monograph on telegraphists’ cramp was written by Cronbach (28), who described 17 cases which he had observed in Berlin.

Eye and facial spasms were a yet different condition. A notable artistic description of cranial dystonia was likely provided circa in 1558 by the vivid painting of an elderly woman made by Pieter Brueghel (29). The first medical description of blepharospasm was given in 1906 by a French ophthalmologist (30). Meige is credited to have described cases of blepharospasm and other cranial dystonias (31). Patients had predominantly symmetric dystonic spasms of facial muscles, sometimes associated with dystonic movements of other midline muscle groups. After the original description, little appeared in the literature until 1972, when there were reports of isolated oromandibular dystonia (32) and oromandibular dystonia with blepharospasm (an association then described with the eponym “Meige’s syndrome”) (33). Marsden later used the expression “Brueghel syndrome” to describe a large series of patients and noted that it usually started in the sixth decade with blepharospasm, oromandibular dystonia, or both. Meige’s syndrome later became the preferred expression (34).

Spasmodic dysphonia was probably first described by Traube in the second volume of his textbook (35) and later by Meige (36) and by Macdonald Critchley, who argued whether to call it “spastic” or “dystonic” dysphonia (37). These two terminologies have been used interchangeably until very recently.

## Lumping Focal Dystonias Together

### Genetic Studies

The first glimpse to a possible connection among different focal dystonia syndromes was the observation that two patients with “spasmodic torticollis” also had “functional spasms” in other body regions, including the right thigh, the upper limb while writing (writer’s cramp) and the left foot (10). A second contribution toward lumping together different types of spasms came by the observations that ablative surgery was helpful in focal and generalized dystonia regardless of its etiology (38, 39).

Zeman et al. (40) summarized very neatly the features of idiopathic dystonia: “The chief symptoms are dystonic postures and dystonic movements. The latter are true hyperkinesias and are characterized by relatively slow, long-sustained, powerful, non-patterned, contorting activities of the axial and appendicular muscles. The muscles most commonly involved are those of the neck, trunk, and proximal portions of extremities. Involvement of unilateral muscle groups often results in bizarre torsion movements, hence the alternative term ‘torsion dystonia’ for the disease.” … “‘Dystonic posture’ is the term used if the end position of a dystonic movement is maintained for any length of time. Eventually this may lead to contracture deformities.” Zeman et al. (40) reviewed the published cases with autosomal dominant or recessive transmission and distinguished these from sporadic ones. They reported a four-generation family with autosomal dominant inheritance and so discussed phenotypic heterogeneity.

“Within this family there is a considerable variability of expressivity of the disease. For instance, V-14 shows symptoms of dystonia in a very mild form, manifested by temporary limping, torticollis and blepharospasm. At times this patient appears almost normal. On the other hand, three of his siblings are totally crippled and helpless. V-10 has neither spontaneous movements nor the typical dystonic posture. Yet, upon performing certain volitional movements, typical dystonic features can be easily elicited. Certainly in cases like these two, one could take the position that such manifestations do not justify the diagnosis of dystonia. Yet, it would be illogical to consider any other diagnosis in view of the fact that grandmother, father, siblings, and one child are so definitely affected by dystonia. Applying ‘Occam’s razor’ of scientific parsimony, it is certainly the most logical conclusion that this family exhibits ‘formes frustes’ as well as full-fledged cases of dystonia. Obviously, the strict diagnostic criteria as set forth by Herz (21) were not applied to the subject cases.” It was then recognized that “idiopathic torsion dystonia” is a genetic disorder with heterogeneous phenomenology, from focal to generalized within a same family.

The following years witnessed the development of stereotactic and functional neurosurgery, which was applied to dystonia as well as to Parkinson’s disease. At the same time, there were attempts to understand and classify the diverse causes of dystonia. Levodopa, the newly discovered treatment for Parkinson’s disease, was also tried in dystonia (41). Denny-Brown (42) did not contribute much to understanding the phenomenology of dystonia, although he attempted to distinguish dystonia occurring in Huntington’s chorea, athetosis, dystonia musculorum deformans, and parkinsonism. He described possible anatomical correlates of each of these dystonia syndromes; he also performed brain lesions in monkeys and called “dystonia” whatever postural phenomenon he could observe, including postural abnormalities associated with spastic hemiplegia (“cortical dystonia”).

In 1966, Jacob A. Brody, Chief of Epidemiology Branch at NINDS, and Irving S. Cooper, Head of Neurologic Surgery at St. Barnabas Hospital, discussed possible epidemiologic studies utilizing the large population of patients with neurological diseases seen at St. Barnabas Hospital over the years. During the course of their talks, Dr. Cooper mentioned that he had operated on approximately 200 patients with torsion dystonia. Dr. Roswell Eldridge, a trained medical geneticist, had just joined Dr. Brody’s staff, and he sensed a fertile ground for a genetic study of this disease. He then gathered a body of information on torsion dystonias, which provided important insights on the various genetic forms of the disease, their clinical presentation, and geographic and ethnic patterns. On January 9, 1970, Dr. Roswell Eldridge convened a conference on the torsion dystonias at NIH in Bethesda, MD, USA.

### Clinical Observations

When he moved from St. Thomas’s Hospital to King’s College in 1970, David Marsden was already interested in the pathophysiology of movement and had studied the physiology of human tremor. In 1973, he gave a lecture on drug treatment of diseases characterized by abnormal movements at a symposium on “Involuntary movements other than parkinsonism” (43). On that occasion, he reported that “chorea (including hemiballism and orofacial dyskinesia) and generalized torsion dystonia may be considered together, for the abnormal movements of both can be reduced by the same groups of drugs, although neither can be cured,” and that “spasmodic torticollis must also be mentioned briefly, for it is a common problem.” He later became fascinated by dystonia, an involuntary movement with irregular features compared to tremor and with unknown pathophysiology.

In his publication dedicated to the review of 42 patients with dystonia, Marsden recognized that “idiopathic torsion dystonia (dystonia musculorum deformans) is a rare and fascinating disease” (44). This paper contains all the elements for considering dystonia a unique disease. He used Herz’s diagnostic criteria (21) and distinguished three types of onset: the commonest being a difficulty to use one or both arms, the second commonest was an abnormality of gait, whereas in a minority of patients, the initial abnormality was confined to the neck and trunk. In half of the patients, the disease progressed to involve all the limbs and trunk, and in the remaining half, the disease was confined to one portion of the body and never became generalized. Marsden still considered cervical dystonia a separate entity and stated: “the characteristic features of torsion dystonia in adults is that it is usually restricted to one part of the body and that it is usually non-progressive. In these respects adult-onset torsion dystonia strikingly resembles isolated spasmodic torticollis, which we deliberately excluded from the study” (44).

**Figure 1:**
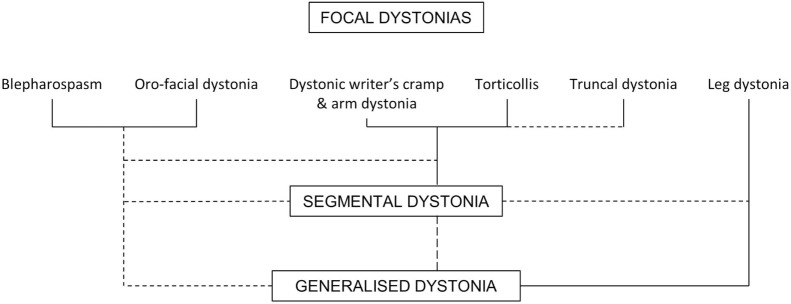
**“Possible interrelations between blepharospasm, oromandibular dystonia, dystonic writer’s cramp, and torticollis/axial dystonia (focal dystonias), and idiopathic torsion dystonia of segmental or generalized type**. Common associations are shown by solid lines, and rare transitions by dashed lines” modified from (47). Marsden’s handwritten schemes can be found also in other publications, such as the Roberg Wartenberg lecture (49) or the Phoenix and Firkin beermats (48).

In 1975, Marsden attended the first international conference on dystonia that was convened in New York by Roswell Eldridge and Stanley Fahn. Compared to the conference held in Bethesda 5 years before, this was called international as the faculty originated also from outside the United States. Stanley Fahn, who was at Columbia University in New York, had seen the daughter of Samuel and Frances Belzberg who was affected by generalized dystonia and consulted with Dr. Eldridge, who had extensively reviewed the genetic epidemiology of dystonia at the 1970 conference (45). The Belzberg family supported the creation of the Dystonia Foundation (today Dystonia Medical Research Foundation) and the 1975 international symposium. Stanley Fahn was particularly interested in the phenomenology and classification of dystonia and defended the view that dystonia was a symptom of several different dystonic syndromes, which he attempted to classify (46). At the same meeting, Marsden reported on “the problem of adult-onset idiopathic torsion dystonia and other isolated dyskinesias in adult life (including blepharospasm, oromandibular dystonia, dystonic writer’s cramp, and torticollis, or axial dystonia)” (47). The slide he showed lumping together blepharospasm, orofacial dystonia, writer’s cramp, torticollis, truncal dystonia, and leg dystonia was quite visionary (Figure 1). He probably drafted it at one of the afterhours meetings with his assistants and fellows at The Phoenix and Firkin pub in Denmark Hill (48).

The founding of the modern concept of dystonia is considered to have occurred exactly 40 years ago, in 1976, when David Marsden published several reports on dystonia (50–53). One of these accounts, in particular, suggested that blepharospasm could be a variant of adult-onset focal dystonia (53). This was a striking notation, not only due to the vivid picturing of *De Gaper*, by Pieter Brueghel the Elderly; but also because blepharospasm had not previously entered the spectrum of dystonia, and was not considered a phenomenology of *formes frustes* or a body part involved in generalized dystonia. Marsden concluded that “(1) blepharospasm and oromandibular dystonia are manifestations of a single illness or syndrome; (2) this is a physical illness, not a manifestation of a psychiatric disorder; (3) this syndrome is related to idiopathic torsion dystonia.”

In the following years at Denmark Hill, the interest in myoclonus melted with that of dystonia and spanned from phenomenology to physiology and experimental animal models. Chorea and dystonia were recognized in patients with Parkinson’s disease (54, 55). Primary writing tremor was described as a condition independent of “myoclonic jerks occurring in dystonia” and of “benign essential tremor” (56).

In 1981, an *ad hoc* committee established by the Research Group on Extrapyramidal Disorders of the World Federation of Neurology, chaired by André Barbeau (including David Marsden, but not Stanley Fahn) proposed that “hyperkinesias” encompassed tremors, tics, myoclonus, chorea, ballism, athetosis, and akathisia (57). Dystonia was listed under “disorders of posture and tone” aside “torsion spasm,” cogwheel phenomenon, hypertonia (encompassing rigidity and Gegehalten), and hypotonia. This classification did not have follow-up. By the same time, in a parallel publication Marsden listed chorea, dystonia, tremor, myoclonus, and tics under the collective heading of dyskinesias, as distinguished from rigid-akinetic syndromes (58). In a celebrated Robert Wartenberg lecture, delivered in April 1981 in front of the American Academy of Neurology, David Marsden summarized his vision of basal ganglia functions by putting together pathophysiology, anatomy, nosology, and phenomenology (49). Around that time, he conceived and crafted along with Stanley Fahn the intellectual and practical infrastructure for the current thinking on dystonia and other movement disorders. Together, they discerned and promulgated the critical clinical characteristics that distinguish dystonia from other involuntary movements (59).

The focal dystonias were then lumped together, as a coherent clinical set encompassing cranial dystonia (including blepharospasm), oromandibular dystonia, spasmodic torticollis, truncal dystonia, writer’s cramp, and other occupational dystonias (60). Specific publications were dedicated to characterize this newly defined large set of organic diseases called dystonias: “spastic dysphonia” (61), writer’s cramp (62), myoclonus dystonia (63), and tardive dystonia (64). At the same time, neurophysiological studies aimed to identify a common pathophysiology for the dystonia syndromes: a quite challenging task (65, 66).

## Current Views

Is dystonia one or many diseases? The lucid effort of lumping together conditions that were previously considered separate diseases has greatly contributed to the modern era of movement disorders but has left unsolved the issue of whether dystonia is to be regarded today as a single disease entity, a syndrome, a collection of physical signs, or as a somehow heterogeneous collection of syndromes. The cases for lumping different dystonias into a unique disease entity, or by the contrary to split them into separate conditions, have been the object of a recent review (67). There is no unique etiology or pathophysiology for different dystonia syndromes and—although a number of features clearly overlap—we have to admit that clinical features, etiology, and pathophysiology are heterogeneous. Dystonia still remains a mysterious condition notwithstanding a dramatic increase in knowledge.

Table 2 summarizes the evolution of observed clinical features of dystonia syndromes. Some observations have been retained over time, the most solid one being the recognition of dystonic movements and postures as the hallmarks features of dystonia, as noted by Herz’s cinematic studies (21). Some observations deal with dystonia in general, while others list features and diagnostic criteria for specific focal dystonia types. These two viewpoints do not necessarily overlap.

**Table 2:** **Features observed in dystonia and diagnostic criteria for different dystonia types have evolved over time**.

Year	Features	Applies to	Reference
1944	Dystonic movements: slow, long-sustained turning movements of the head and trunk and rotations of the upper or lower extremities. Show a still more pronounced excess of tension, which prevails over the excess of motion. Dystonic postures: peculiar positions, which occur in various combinations. Show the influence of excess of tension in almost pure form.	Generalized dystonia (symptomatology)	(21)

1956	Involuntary tonic but spasmodic bilateral contraction of the orbicularis oculi, in which the spasm of the eyelids may last from several seconds to several minutes, with periods of relaxation of varying length interspersed	Blepharospasm	(68)

1959	The chief symptoms are dystonic postures and dystonic movements The muscles most commonly involved are those of the neck, trunk, and proximal portions of extremities	Dystonia in general	(40)

1974	An illness characterized by the development of dystonic movements and postures	Generalized dystonia (idiopathic torsion dystonia, dystonia musculorum deformans)	(44)

1982	Definitions Simple cramp: difficulty performing only one specific task Dystonic cramp: muscle spasms in several tasks Progressive cramp: increasing difficulty in performing new tasks	Upper limb dystonia (writer’s cramp, typist’s cramp, pianist’s cramp)	(62)
	Associated neurological signs Tremor Increased limb tone Decreased arm swing Dystonic posture		

1984	Description of the varied phenomenology of “rapid” dystonic movements occurring in different body regions: upper face, lower face, jaw, pharynx, tongue, neck, arm, trunk, leg, segmental, generalized (in addition to the description of slow dystonic movements by Herz) (21)	Dystonic movements	(69)

1988	Elements identified by physiologic investigation that are indicative of impaired motor control in dystonia Co-contraction of antagonist muscles Prolongation of EMG bursts Tremor Lack of selectivity in attempts to perform independent finger movements Failure of willed activity to occur	Upper limb dystonia	(70)

1988	A syndrome of sustained muscle contractions, frequently causing twisting and repetitive movements, or abnormal postures	Dystonia in general	(71)

1988	Repetitive involuntary sustained contractions of orbicularis oculi Increased blinking frequently is the first sign of blepharospasm	Blepharospasm	(72)

1991	Presenting symptoms: pulling in the neck (59%), head tremor (14%), neck pain (17%), head jerking (11%), neck stiffness/tightness (7%), a combination of any two of these symptoms (18%)	Cervical dystonia	(73)

1991	Pain is a specific feature of cervical dystonia	Cervical dystonia	(74)

1994	In addition or as an alternative to typical spasm of the orbicularis oculi, there may be failure to voluntarily open the eyes with no apparent spasm of the orbicularis oculi (sometimes called “apraxia” of eyelid opening)	Blepharospasm	(75)

1997	Dystonia: dystonic posturing and slow torsion movements are evident to all the examiners. Blepharospasm: all the examiners find at least two prolonged spasms of the orbicularis oculi muscles. Writer’s cramp: at least three of the following occur: (1) a progressive change in handwriting, (2) a progressive change of handgrip, (3) hand posturing and increased pressure on the sheet during handwriting, (4) abnormal contraction of brachia! or antebrachial muscles during handwriting, and (5) hand posturing associated with abnormal proximal movements of the arm or shoulder while writing.	Dystonia syndromes (definite diagnosis for family study)	(76)

2000	Distinguishing clinical features of dystonia Speed of contractions may be slow or rapid, but at the peak of movement, it is sustained. Contractions almost always have a consistent directional or posture assuming character. Predictably involves one or more body regions. Usually aggravated during voluntary movement (action dystonia) and may only be present with specific actions (e.g., writing); alternatively certain actions may improve dystonia—known as paradoxical dystonia (e.g., speaking often improves oromandibular dystonia). May progress to involve more body regions and more actions, eventually involving rest. Usually varies with changes in posture. Worse with stress, fatigue; better with rest, sleep, and hypnosis. Sensory tricks (tactile or proprioceptive maneuver) lessen contractions (touching cheek improves torticollis).	Dystonia in general	(77)

2002	Definite dystonia: characteristic overt twisting or directional movements and postures that are consistently present. Probable dystonia: postures or movements suggestive of dystonia that are insufficient in intensity or consistency to merit classification as definite (e.g., excessively tense and labored writing with minimal posturing, flurries of blinking, but no episodes of sustained closure, mild or intermittent head deviation). Possible dystonia: muscle contractions not considered abnormal but remotely suggestive of dystonia (e.g., unusual hand grip with mild excess hand tension but normal flowing handwriting, increased blinking with no flurries or sustained contractions, clumsy rapid feet movements with intermittent overflow toe posturing). Scoliosis and regular tremor (i.e., without sustained directionality) were not considered signs of dystonia for any category	Dystonia signs and symptoms observed in different subjects of affected families	(78)

2003	In dystonic hypertonia, all of the following are expected Resistance to externally imposed joint movement is present at very low speeds of movement, does not depend on imposed speed, and does not exhibit a speed or angle threshold. Simultaneous co-contraction of agonists and antagonists may occur, and this is reflected in an immediate resistance to a rapid reversal of the direction of movement about a joint. The limb tends to return toward a fixed involuntary posture, and when symptoms are severe, the limb tends to move toward extremes of joint angles. Hypertonia is triggered or worsened by voluntary attempts at movement or posture of the affected and other body parts and may be strongly dependent on the particular movement or posture attempted or the activity of distant muscle groups. The pattern as well as the magnitude of involuntary muscle activity varies with arousal, emotional and behavioral state, tactile contact, or attempted task. There is no other detected spinal cord or peripheral neuromuscular pathology causing tonic muscle activation at rest.	Dystonic hypertonia in children	(79)

2004	Clinical diagnostic criteria Identified kinesigenic trigger for the attacks Short duration of attacks (1 min) No loss of consciousness or pain during attacks Exclusion of other organic diseases and normal neurologic examination Control of attacks with phenytoin or carbamazepine, if tried Age at onset between 1 and 20 years, if no family history of PKD	Paroxysmal kinesigenic dyskinesias	(80)

2006	Dystonia is a movement disorder characterized by patterned, directional, and often sustained muscle contractions that produce abnormal postures or repetitive movements	Dystonia in general	(81)

2006	Blink rate at rest >27 blinks/min and higher than blink rate during conversation suggests a diagnosis of blepharospasm, whereas blink rate during conversation higher that blink rate at rest suggests against such diagnosis	Blepharospasm	(82)

2008 (and 2009)	Features of “ipsilateral overflow” and “contralateral overflow,” as distinct from features of “mirror dystonia”	Upper limb dystonia	(83, 84)

2009 (and 2016)	Dystonic postures: a body part is flexed or twisted along its longitudinal axis; slowness and clumsiness for skilled movements are associated with sensation of rigidity and traction in the affected part. Dystonic movements: either fast or slow; tremor is a feature of dystonic movements and may appear as isolated tremor; movements are repetitive and patterned (i.e., consistent and predictable) or twisting, and often sustained at their peak to lessen gradually in a preferred posture (usually opposite to the direction of movement). Gestes antagonistes (sensory tricks): voluntary actions performed by patients that reduce or abolish the abnormal posture or the dystonic movements. Mirror dystonia: a unilateral posture or movement with same or similar characteristics to the patient’s dystonia that can be elicited, usually in the more severely affected side, when contralateral movements or actions are performed. Overflow dystonia: an unintentional muscle contraction accompanying the most prominent dystonic movement, but in an anatomically distinct neighboring body region.	Dystonia (physical signs) excluding cranial and laryngeal forms	(85, 86)

2011	Specific criteria: observation of involuntary bilateral increased blinking with intermittent eye spasms Patients were also asked about photophobia, dry eyes, and sensory tricks. If present, these symptoms were helpful to make the diagnosis; however, they were not deemed essential	Blepharospasm	(87)

2013	Dystonia is a movement disorder characterized by sustained or intermittent muscle contractions causing abnormal, often repetitive, movements, postures, or both. Dystonic movements are typically patterned, twisting, and may be tremulous. Dystonia is often initiated or worsened by voluntary action and associated with overflow muscle activation	Dystonia in general	(88)

2013	Stereotyped, bilateral, and synchronous orbicularis oculi spasms inducing narrowing/closure of the eyelids Effective sensory trick Increased blinking	Blepharospasm	(89)

2014	List of sensory tricks observed in different dystonia types	Different dystonia types	(90)

*This table lists diagnostic criteria and clinical features that have been proposed to be specific for different dystonia types. Clinical series that did not specifically mention diagnostic criteria for dystonia, classifications schemes, and rating tools are not listed here*.

Ten papers considered the general features of dystonia, defined as a disease, a syndrome, or a collection of physical signs (Table 2). Herz (21) was the first to define diagnostic criteria for dystonia that have been updated in recent years by recognizing five main physical signs of dystonia (85, 86) that can be reliably recognized when the limbs or the neck/trunk are affected. By contrast, these features are difficult to assess in patients with blepharospasm, which has a different phenomenology, or spasmodic dysphonia, where the abnormal movements are hard to see. The five physical signs of dystonia encompass dystonic postures, dystonic movements, tricks/gestes, mirror dystonia, and overflow. When several of these signs occur together in the same patient, a diagnosis of dystonia can be reliably made. As for other medical diagnoses, not all the physical signs have to occur simultaneously, but they need to be in sufficient number to provide a strong diagnostic clue. Some of the general diagnostic criteria for dystonia have been developed for the study of affected families using linkage studies, where the affected or non-affected status provided a discriminant variable (76, 78). In these families, focal phenotypes were considered alternative phenotypes or *formes frustes* of a same genetic condition.

Six publications defined the features and diagnostic criteria of blepharospasm. This focal form has different features from dystonia affecting the limbs or trunk. Diagnostic criteria for blepharospasm have been proposed only recently, and their specificity is a matter of discussion (89). Four publications reported diagnostic features of upper limb dystonia, and two (unlisted) studied assessed lower limb dystonia without indicating diagnostic criteria (91, 92). It is interesting to note that very few publications have tried to list diagnostic features of cervical dystonia, which is still diagnosed based on clinical experience and often considered an easy diagnostic task.

**Figure 2:**
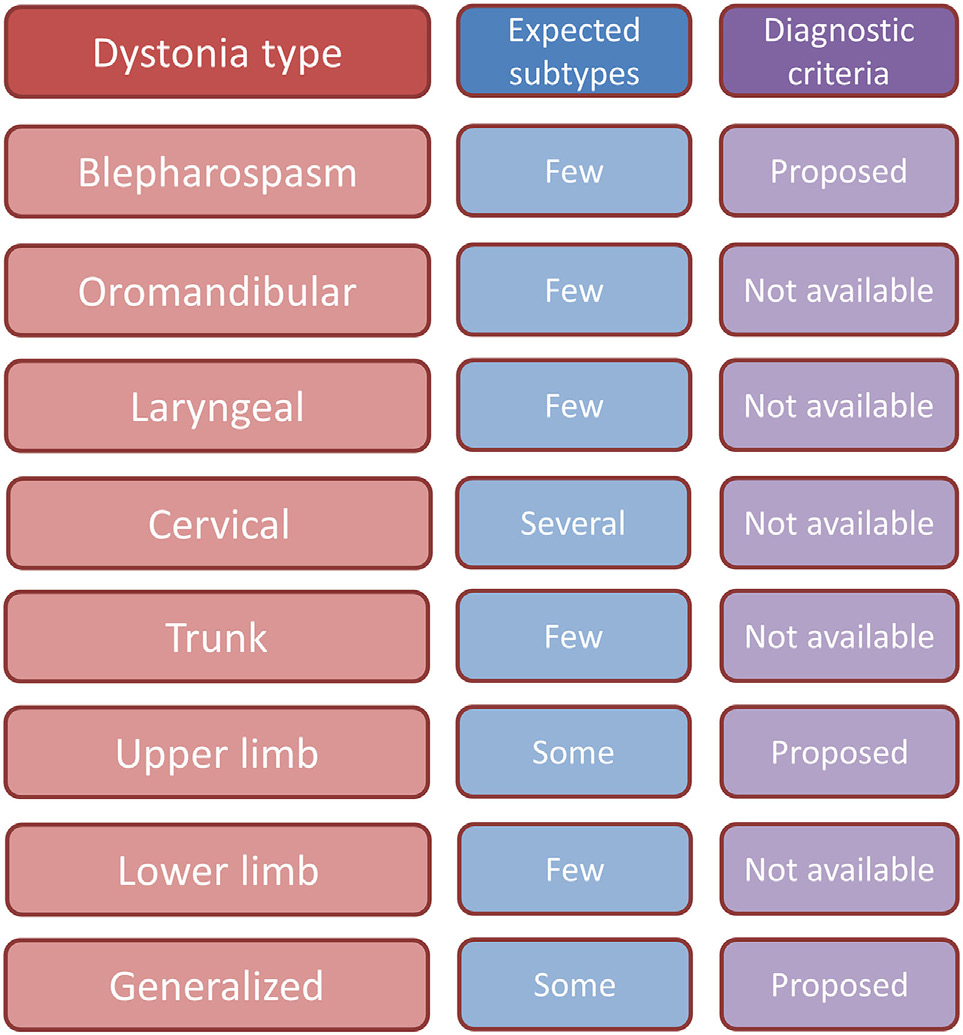
**Synopsis table of expected clinical subtypes and status of proposed diagnostic criteria for different dystonias**.

The swing is currently moving back toward recognizing the specific identity of focal dystonia syndromes. A Delphi method approach is being used by the Movement Disorders Society Dystonia Task Force to identify specific criteria for blepharospasm and for cervical dystonia. In the time to come, general diagnostic criteria for dystonia as a whole and specific criteria for focal dystonia syndromes will probably coexist. Clinical trials on focal dystonias require the harmonic implementation of well-defined criteria in multicentric settings. A currently unmet need is the characterization of clinical subtypes of dystonias and the identification of diagnostic criteria for each of them (Figure 2).

Based on current knowledge, it remains hard to accept dystonia as a single disease: nosologically, it is a collection of syndromes (93); phenomenologically, it is a collection of physical signs (85). However, there is no combination of physical signs that accommodates for all focal and generalized dystonia types: cranial and laryngeal dystonia have evidently a different phenomenology from limb and trunk dystonia. The physical signs of dystonia may apply to cervical and limb dystonia syndromes and may be used for the purpose of clinical diagnosis. By contrast, specific sets of clinical features and related diagnostic criteria still need to be developed for cranial and laryngeal dystonias.

## Author Contributions

The author confirms being the sole contributor of this work and approved it for publication.

## Conflict of Interest Statement

The author declares that the research was conducted in the absence of any commercial or financial relationships that could be construed as a potential conflict of interest.
